# Mortality among People with Severe Mental Disorders Who Reach Old Age: A Longitudinal Study of a Community-Representative Sample of 37892 Men

**DOI:** 10.1371/journal.pone.0111882

**Published:** 2014-10-31

**Authors:** Osvaldo P. Almeida, Graeme J. Hankey, Bu B. Yeap, Jonathan Golledge, Paul E. Norman, Leon Flicker

**Affiliations:** 1 School of Psychiatry & Clinical Neurosciences, University of Western Australia, Perth, Australia; 2 WA Centre for Health & Ageing, Centre for Medical Research, Perth, Australia; 3 Department of Psychiatry, Royal Perth Hospital, Perth, Australia; 4 School of Medicine and Pharmacology, University of Western Australia, Perth, Australia; 5 Department of Neurology, Sir Charles Gairdner Hospital, Perth, Australia; 6 Department of Endocrinology, Fremantle Hospital, Fremantle, Australia; 7 Queensland Research Centre for Peripheral Vascular Disease, School of Medicine and Dentistry, James Cook University, Townsville, Australia; 8 Department of Vascular and Endovascular Surgery, The Townsville Hospital, Townsville, Australia; 9 School of Surgery, University of Western Australia, Perth, Australia; 10 Department of Geriatric Medicine, Royal Perth Hospital, Perth, Australia; The Nathan Kline Institute, United States of America

## Abstract

**Background:**

Severe mental illnesses are leading causes of disability worldwide. Their prevalence declines with age, possibly due to premature death. It is unclear, however, if people with severe mental disorders who reach older age still have lower life expectancy compared with their peers and if their causes of death differ.

**Methods and Findings:**

Cohort study of a community-representative sample of 37892 Australian men aged 65–85 years in 1996–1998. Follow up was censored on the 31st December 2010. Lifetime prevalence of schizophrenia spectrum, bipolar, depressive and alcohol-induced disorder was established through record linkage. A subsample of 12136 consented to a face-to-face assessment of sociodemographic, lifestyle and clinical variables. Information about causes of death was retrieved from the Australian Death Registry. The prevalence of schizophrenia spectrum, bipolar, depressive and alcohol-induced disorders was 1.2%, 0.3%, 2.5% and 1.8%. The mortality hazard for men with a severe mental disorder was 2.3 and their life expectancy was reduced by 3 years. Mortality rates increased with age, but the gap between men with and without severe mental disorders was not attenuated by age. Cardiovascular diseases and cancer were the most frequent causes of death. The excess mortality associated with severe mental disorders could not be explained by measured sociodemographic, lifestyle or clinical variables.

**Conclusions:**

The excess mortality associated with severe mental disorders persists in later life, and the causes of death of younger and older people with severe mental disorders are similar. Hazardous lifestyle choices, suboptimal access to health care, poor compliance with treatments, and greater severity of medical comorbidities may all contribute to this increased mortality. Unlike young adults, most older people will visit their primary care physician at least once a year, offering health professionals an opportunity to intervene in order to minimise the harms associated with severe mental disorders.

## Introduction

Mental and substance use disorders are leading causes of years lived with disability worldwide [1]. There is also evidence that the life expectancy of people with severe mental illnesses is ten to fifteen years lower than that of the general population [2], mainly because of a high number of deaths due to cardiovascular events (myocardial infarctions and strokes), respiratory diseases and suicide [3], [4], [5], [6]. While suicide could be considered a direct consequence of severe mental illness [7], the underlying reasons for the excessive mortality due to cardiovascular and respiratory diseases are less clear. Unhealthy lifestyle practices commonly associated with psychiatric disorders, such as smoking, might play a role [8], [9], [10], [11], as may some medications used to treat these conditions. Second generation antipsychotics have been associated with the metabolic syndrome and cardiovascular events [12], certain antidepressants with cardiovascular complications [13], and anxiolitics and hypnotics with accidental injuries and premature death [14].

Premature mortality may explain, at least in part, the lower prevalence of severe mental disorders such as schizophrenia, mood and alcohol-induced disorders, in late compared with early adult life [15], [16], as the number of new older people affected is lower than the number of prevalent cases who die early. Consequently, older people with severe mental disorders consist of a group of survivors and a relatively small number of new cases [17]. Old survivors could, conceivably, have had a more benign course of illness or healthier lifestyle than those who died early, whereas new cases would have had only limited exposure to the potentially detrimental effects associated with their illness. Thus, there may be no excess mortality among people with severe mental disorders who reach old age. Clarifying whether or not this is the case is important because the number of older people living with severe mental disorders will continue to increase as the population ages [18], and policy makers need to know whether to target this population with interventions that promote health and increase survival.

The Health In Men Study (HIMS) is a longitudinal investigation of a community-representative sample of about 38000 men aged 65–85 years at study entry. Past mental health history was retrieved via health record linkage and mortality data monitored until the end of 2010 [19]. These data were used to determine: (1) the prevalence of severe mental disorders in older men at the start of the study, (2) the 14-year cause-specific mortality of older men with and without severe mental disorders, (3) differences in the life expectancy of older men with and without severe mental disorders, (4) the clinical and lifestyle characteristics of participants with and without severe mental disorders who consent to assessment. We hypothesised that: (1) the prevalence of alcohol-induced, mood and schizophrenia spectrum disorders (severe mental disorders) would be lower than that reported for younger adults, (2) the mortality hazard, causes of death and life expectancy of older men with and without severe mental disorders would be similar, and that (3) men with and without severe mental disorders would have similar lifestyle and clinical characteristics.

## Methods

### Study design and setting

HIMS is a cohort study of a community-representative sample of older Western Australian men that started in 1996–1998. Follow up data were censored on the 31st December 2010.

### Participants

Men aged 65–85 years were identified using an electronic copy of the Western Australian electoral roll (enrolment to vote is compulsory for all Australian adults). We received a list with the contact details of 49801 men. Of those, 1839 had already died at the time the study started and another 9482 did not receive an invitation to participate because they were living outside the immediate Perth metropolitan region. Of the remaining 38480 men, 588 were excluded from further follow up because they had a recorded diagnosis of dementia (International Classification of Diseases 9th edition, ICD-9 code 290). Hence, the study sample for this study consisted of 37892 older men free of dementia at study entry who were recruited between the 2nd April 1996 and the 18th November 1998. Of these, a random sample of 18968 received an invitation to complete a face-to-face assessment: 12136 accepted.

This study followed the principles of the Declaration of Helsinki and was approved by the Ethics Committees of the University of Western Australia and of the Department of Health of Western Australia. The 12136 men who completed the face-to-face assessment provided written informed consent to participate. The remaining 25756 men were not asked to provide written informed consent. For this reason, their data were anonymised and de-identified by the Data Linkage Unit of the Department of Health of Western Australia prior analysis. The procedures for data extraction and analysis followed the regulations for privacy and security of Western Australia (http://www.datalinkage-wa.org.au/privacy-and-security), and were approved by the Human Research Ethics Committee of the Department of Health and by the Legal Data Custodian of the Department of Health of Western Australia.

### Healthy participant bias

This study used exposure and outcome data for the entire population, so that no participation bias is involved. However, only 12136 of the 18968 participants invited completed the assessment for HIMS. For this reason, we examined the prevalence of mental disorders and mortality among non-invited men (n = 18924), HIMS participants who completed the assessment (n = 12136), and invited men who did not provide a response to our invitation (n = 6832).

### Outcomes

The primary outcome of interest in this study was all-cause mortality, which we measured using the Western Australian Data Linkage System (WADLS).[19] WADLS retrieves information from the Australian Bureau of Statistics about all deaths in Australia, including information about the reported causes of death (http://www.abs.gov.au/ausstats/abs@.nsf/mf/3303.0). We used the International Classification of Diseases codes (ICD-9 until 30/06/2008 and ICD-10 from 01/07/2008) to group the causes of mortality into the following categories: infections (ICD-9 codes ranging from 001 to 139; ICD-10 codes starting with ‘A’ or ‘B’), cancers (ICD-9 codes ranging from 150 to 165, 185 to 192, 200 to 208; ICD-10 codes starting with ‘C’, D00 and D48), cardiovascular diseases (ICD-9 codes 410 to 414, 430 to 438; ICD-10 codes starting with ‘I’), chronic respiratory diseases (ICD-9 codes 490 to 496; ICD-10 codes starting with ‘J’), substance-induced or mental diseases (ICD-9 codes 290 to 329; ICD-10 codes starting with ‘F’), diseases of the nervous system (ICD-9 codes 330–339; ICD-10 codes starting with ‘G’), suicide (ICD-9 codes E950 to E959, E960 to E969; ICD-10 codes X60 to X84), and accidents (ICD-9 codes E800 to E899, E910; ICD-10 codes starting with ‘V’ or W00 to W19). Deaths due to other causes were placed in a separate group.

### Exposures at study entry

We retrieved information about past diagnosis of mental disorders from the WADLS, which connects together all death records, acute hospital admissions (including psychiatry), hospital movements, cancer registry and psychiatric outpatient contacts. The mental health records go back to 1966 (i.e., 30 years before the starting date for this study) [19]. In this study, we were interested in the effect of severe mental disorders that would require input from specialist mental health services: schizophrenia spectrum disorders, bipolar disorder, depressive disorder, and alcohol-induced disorders. Schizophrenia spectrum disorders included schizophrenia, schizophreniform and schizoaffective disorders (ICD-9 codes 295), delusional disorders (ICD-9 codes 297), other schizophrenia spectrum disorders (ICD-9 codes 298.8 and 298.9), and schizotypal disorder (ICD-9 code 301.22). A diagnosis of bipolar disorder was assigned to men who had a recorded ICD-9 code 296.0, 296.4, 296.5, 296.6, 296.7, and 296.80 or 296.89. Men with the following ICD-9 codes were deemed to have had depression before joining the study: 296.2, 296.3, 296.82, 296.90, 298.0, 311. Men with a past recorded ICD-9 code 291 or 303 were identified as having a alcohol-induced disorder. The diagnosis of schizophrenia spectrum disorder took precedence over all other diagnosis, followed by bipolar disorder, depressive disorder and, finally, alcohol-induced disorder. We grouped all other men under the label ‘no severe mental disorder’. Previous studies have shown that WADLS yields accurate diagnoses for severe mental disorders [20].

### Other study measures at study entry and at the face-to-face assessment

We calculated the age of participants as the difference, in years, between the date of study entry and the date of birth. We then grouped men in 5-year blocks: 65–69, 70–74, 75–79 and 80 or more years. In addition, the 12136 men who completed the clinical assessment provided information about their place of birth (Australia or overseas), marital status, education (high school completed or not), and past self-reported medical history of strokes, angina or heart attacks or bypass surgery, bronchitis or asthma, diabetes and hypertension. We also measured the blood pressure of participants using standardised procedures, and reclassified as hypertensive men with a systolic blood pressure ≥140 mmHg or diastolic blood pressure>90 mmHg.

We measured the height of participants in centimetres (without shoes) and their weight in Kilograms (to 0.2 Kg, light clothing). The body mass index (BMI) was calculated in Kg/m^2^ and was used to assign men to the following groups: underweight (BMI<18.5), normal (18.5≤BMI<25), overweight (25≤BMI<30) and obese (BMI≥30). We also asked men how many minutes they spent in a usual week doing vigorous exercise (that made them breathe hard or puff and pant) and considered those who reported 150 minutes or more of weekly activity to be physically active. We inquired whether they had ever smoked (yes/no) and to men who answered ‘yes’, we asked whether they were still smoking (yes/no). We used this information to group men into never smokers, past smokers and current smokers. Finally, participants answered the following questions about their use of alcohol: ‘Have you ever drunk alcohol?’ (yes/no), ‘Have you drunk alcohol in the last year?’ (yes/no), ‘If yes, how many standard drinks do you have each day in a usual week?’ A standard drink is equivalent to 10 grams of alcohol. We provided guidelines to assist participants calculate the number of drinks consumed.

### Study size

A study of this size (n = 37892) would be expected to include at least 150 men with schizophrenia spectrum disorder [21], 150 with bipolar disorder [22], and 750 each with major depression and alcohol-induced disorders [23], [24]. We would expect about 50% of older men without a severe mental disorder to die during follow up. If an additional 5% of those with a severe mental disorder die during the same period, this study would have over 95% power to declare this difference as significant for a two-tailed alpha of 5%.

### Statistical methods

We used the statistical package Stata/IC 13.1 to manage and analyse the data (StataCorp LP, 2013). Descriptive statistics summarised categorical data as count and proportions (%), and continuous variables as mean, range, and standard deviation of the mean (SD). Of the 37892 men included in the study, a random sample of 18,968 received an invitation for assessment, and 12136 attended their appointment. Details about the selection procedure have been described elsewhere [25].

We used the proportion command of Stata to estimate the prevalence of severe mental disorders (95% confidence intervals were calculated using a logit transformation that allows for the estimation of the standard error of the proportion). We subsequently employed Cox regression to estimate the mortality hazard ratio (HR) of older men with past recorded history of severe mental disorders, and set the origin on the participants' date of birth, the beginning of follow up as the date of randomisation, and the date of death as the time of the event. Data were censored on the 31st December 2010. Survival data were plotted using the Kaplan-Meier survivor function. We calculated the mortality rate of people with and without severe mental disorders per-thousand person-years using the strate command of Stata. A similar Cox regression model was used to estimate the mortality hazard of the 12136 men who completed the assessment for HIMS.

Analysis of the risk of specific causes of death according to the four severe mental disorder groups (schizophrenia spectrum, bipolar, depression and alcohol-induced) was based on competing risks regression models. These models investigate the event of interest (e.g., death from cardiovascular disease) when competing events are present and impede the occurrence of the event of interest (e.g., death by cancer or by a respiratory disease, etc.). The risk estimates, in this case, are expressed as sub-hazard ratio (SHR).

We used Pearson's chi-square statistic to compare the distribution of severe mental disorders, age, and death during the subsequent follow up of these three samples (i.e., controls not invited, invited participants who underwent assessment, and invited participants who did not respond to our invitation for assessment). The age distribution of these three groups was also compared using oneway analysis of variance. Logistic regression was applied to determine the odds ratio (OR) of having a severe mental disorder of HIMS participants and non-responders compared with controls, as well as their odds of death.

We calculated the 95% confidence interval (95%CI) for all risk estimates, rates, differences and proportions. Alpha was set at 0.05 and all testes reported were two-tailed.

## Results

The study sample consisted of 37892 men aged 65 to 85 years at study entry (mean = 72.4, SD = 4.6). Of these, 444 had a recorded diagnosis in WADLS of schizophrenia spectrum (1.2%, 95%CI = 1.1%, 1.3%), 101 of bipolar (0.3%, 95%CI = 0.2%, 0.3%), 958 of depression (2.5%, 95%CI = 2.4%, 2.7%), and 698 of alcohol-induced disorder (1.8%, 95%CI = 1.7%, 2.0%). Oneway analysis of variance showed that men with a past diagnosis of alcohol-induced disorder were 0.5, 1.2 and 1.8 years younger than men without a severe mental disorder, schizophrenia spectrum and depression respectively (p<0.05 after Scheffe correction for multiple comparisons). Men with depression were 1.3, and 2.0 years older than their counterparts with no diagnosis and with diagnosis of bipolar disorder (p>0.05 after Scheffe correction for multiple comparisons).

**Figure 1:**
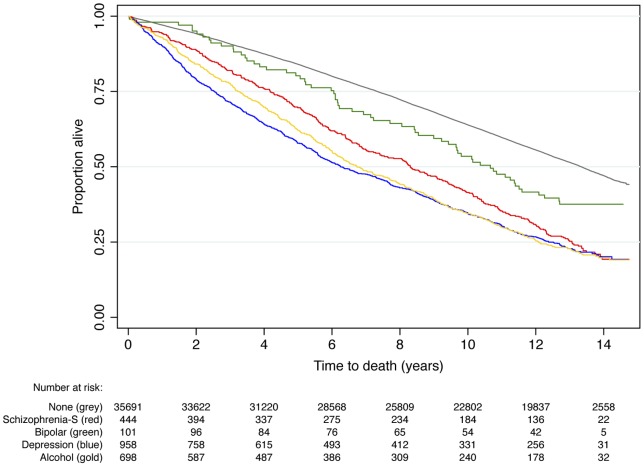
Survival over a follow up period of up to 14.7 years of a community-representative sample of older men with and without serious mental health disorders. The age-adjusted mortality hazard was 2.0 (95%CI = 1.8,2.2), 1.5 (95%CI = 1.2,1.9), 2.3 (95%CI = 2.1,2.5) and 2.6 (95%CI = 2.4,2.8) for men with a past diagnosis of schizophrenia spectrum disorder (Schizophrenia-S), bipolar disorder, depression and alcohol-induced disorders.

By 31st December 2010, 19644 men had died (51.8%), of whom 343 had a schizophrenia spectrum disorder, 63 bipolar, 747 depression and 550 an alcohol-induced disorder. Figure 1 shows the proportion of participants alive during follow up according to the presence of a severe mental disorder. The age-adjusted mortality hazard ratio for men with a severe mental disorder was 2.3 (95%CI = 2.2, 2.4) and their life expectancy was reduced by 2.8 years (95%CI = 2.6, 3.0) compared with their peers. Age-adjusted life expectancy was reduced, on average, 2.0 (95%CI = 1.6, 2.3), 1.1 (95%CI = 0.4, 1.9), 2.8 (95%CI = 2.6, 3.1) and 3.1 (2.8, 3.4) years for men with schizophrenia spectrum, bipolar, depressive and alcohol-induced disorder, respectively. We then completed a sensitivity analysis by excluding from the sample men who died within the first two years of follow up: the mortality hazard associated with any severe mental disorder remained largely unchanged (HR = 2.1, 95%CI = 2.0, 2.2 after the sensitivity analysis).

**Figure 2:**
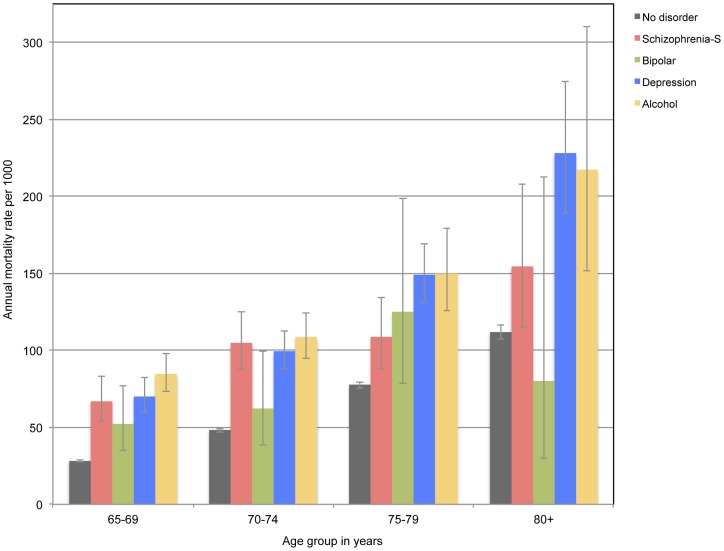
The bars show the mortality rate per 1000 person-years for older men with and without severe mental disorders according to their age group at the time of enrollment. The whiskers indicate the 95% confidence limits of the mean rate. Schizophrenia-S: schizophrenia spectrum disorders. Mean time to event (death or end of follow up) was 10.1 years (standard deviation  = 4.2; range 1 day to 14.7 years).

We stratified participants by age groups (65–69, 70–74, 75–79, 80+) to ascertain their mortality rate. Figure 2 shows the results of these analyses according to the presence of a severe mental disorder before randomisation. The annual mortality rate of men increased with increasing age and was consistently higher amongst those with than without a severe mental disorder. The mortality rate estimate for men with bipolar disorder was imprecise for those aged over 80 years because there were only 6 people in this group.

Table 1 shows the causes of death of participants with and without a severe mental disorder. Men with past diagnosis of schizophrenia spectrum disorders showed excess mortality due to infections, cardiovascular diseases, chronic respiratory diseases, substance-induced or mental disorders, diseases of the nervous system and other. These were no suicides in this group. Men with past diagnosis of bipolar disorder died more frequently than controls of chronic respiratory diseases, suicide, and mental disorders (including substance-induced). Men with depression showed evidence of excess mortality due to cancer, cardiovascular events, chronic respiratory diseases and diseases of the central nervous system, as well as suicide and other causes of death. Finally, men with a past diagnosis of alcohol-induced disorder died more frequently than men without a severe mental disorder of infections, cancer, cardiovascular and respiratory diseases.

**Table 1:** Causes of death over a 14-year period among older men with and without severe mental disorders.

	No severe mental disorder	Schizophrenia-S	Bipolar	Depression	Alcohol
	N = 35691	N = 444	N = 101	N = 958	N = 698
	n (%)	n (%)	n (%)	n (%)	n (%)
Causes of death:	1 (Reference)	SHR (95%CI)	SHR (95%CI)	SHR (95%CI)	SHR (95%CI)
Alive	17750 (49.7)	101 (22.7)	38 (37.6)	211 (22.0)	148 (21.2)
Infection	222 (0.6)	7 (1.6)	0	9 (0.9)	9 (1.3)
		**2.4 (1.1, 5.1)**	–	1.5 (0.8, 2.9)	**2.0 (1.0, 3.9)**
Cancer	5976 (16.7)	61 (13.7)	11 (10.9)	185 (19.3)	143 (20.5)
		0.8 (0.6, 1.0)	0.6 (0.3, 1.1)	**1.2 (1.0, 1.4)**	**1.2 (1.0, 1.5)**
Cardiovascular diseases	6160 (17.3)	118 (26.6)	23 (22.8)	254 (26.5)	185 (26.5)
		**1.6 (1.3, 1.9)**	1.4 (0.9, 2.1)	**1.6 (1.4, 1.9)**	**1.6 (1.4, 1.9)**
Chronic respiratory diseases	1634 (4.6)	44 (9.9)	10 (9.9)	102 (10.6)	86 (12.3)
		**2.1 (1.6, 2.9)**	**2.2 (1.2, 4.1)**	**2.4 (2.0, 2.9)**	**2.8 (2.2, 3.4)**
Substance induced, mental disorders	432 (1.2)	22 (4.9)	5 (4.9)	14 (1.5)	13 (1.9)
		**3.7 (2.4, 5.7)**	**4.2 (1.7, 10.4)**	1.1 (0.7, 1.9)	1.5 (0.9, 2.6)
Nervous system, including dementia	702 (2.0)	33 (7.4)	1 (1.0)	44 (4.6)	14 (2.0)
		**3.7 (2.6, 5.2)**	0.5 (0.1, 3.5)	**2.3 (1.7, 3.1)**	1.0 (0.6, 1.7)
Suicide	78 (0.2)	0	2 (2.0)	7 (0.7)	1 (0.1)
		**–**	**8.6 (2.1, 35.0)**	**3.6 (1.7, 7.9)**	0.6 (0.1, 4.4)
Accidents	249 (0.7)	5 (1.1)	1 (1.0)	7 (0.7)	7 (1.0)
		1.5 (0.6, 3.7)	1.4 (0.2, 10.0)	1.0 (0.5, 2.2)	1.4 (0.7, 2.9)
Other	2488 (7.0)	53 (11.9)	10 (9.9)	125 (13.0)	92 (13.2)
		**1.7 (1.3, 2.2)**	1.4 (0.8, 2.7)	**2.0 (1.6, 2.4)**	**1.9 (1.5, 2.4)**

Schizophrenia-S: schizophrenia spectrum disorders.

SHR: sub-hazard ratio derived from competing-risks regression. 95%CI: 95% confidence interval of the sub-hazard ratio.

Bold print denotes statistically significant association.

Table 2 shows the characteristics of men according to their randomisation assignment: not-invited and invited HIMS participants. Men who joined HIMS were 0.4 and 1.1 years younger than non-invited participants and those who did not respond. In addition, HIMS non-responders were 0.7 years older than men who were not invited (p<0.05 for all analyses, after Scheffe correction for multiple comparisons). There was also an excess of men with severe mental disorders among non-responders than among non-invited men or those included in HIMS. Compared with non-invited men, men included in HIMS had a lower odds of severe mental disorder (OR = 0.7, 95%CI = 0.6, 0.8), whereas non-responder had 43% greater odds of having a severe mental disorder (OR = 1.4, 95%CI = 1.3, 1.6). Similarly, HIMS participants had lower odds of death than non-invited men (OR = 0.8, 95%CI = 0.7,0.8), whereas non-responders had higher chance of dying (OR = 1.5, 95%CI = 1.4, 1.5). Compared with non-invited men, HIMS participants lived 0.7 years longer (95%CI = 0.6, 0.8), whereas non-responders died 1.1 (95%CI = 1.0, 1.2) years earlier than non-invited men and 1.8 (95%CI = 1.6, 1.9) years earlier than HIMS participants.

**Table 2:** Basic characteristics of participants according to their random assignment to the control or Health In Men Study (HIMS) group.

		Controls	HIMS			
			Included	No response		
		N = 18924	N = 12136	N = 6832		
		n (%)	n (%)	n (%)	Chi-square statistic (df)	p-value
Age in years:	65–69	6768 (35.8)	4731 (39.0)	2150 (31.5)	264.94 (4)	<0.001
	70–74	6549 (34.6)	4210 (34.7)	2213 (32.4)		
	75–79	4224 (22.3)	2502 (20.6)	1748 (25.6)		
	80+	1383 (7.3)	693 (5.7)	721 (10.5)		
Schizophrenia spectrum		228 (1.2)	97 (0.8)	119 (1.7)	132.54 (8)	<0.001
Bipolar disorder		49 (0.3)	28 (0.2)	24 (0.3)		
Depressive disorder		479 (2.5)	236 (1.9)	243 (3.6)		
Alcohol induced disorder		366 (1.9)	154 (1.3)	178 (2.6)		
Died during follow up		9849 (52.0)	5608 (46.2)	4187 (61.3)	398.54 (2)	<0.001

df: number of degrees of freedom.

HIMS participants were, on average, 0.4 years younger than controls (95% confidence interval of the mean difference, 95%CI = 0.3,0.5; p<0.001) and 1.1 (95%CI = 0.9,1.2; p<0.001) years younger than men who did not respond to the invitation to join the study. Non-invited men were 0.7 years (95%CI = 0.5,0.8; p<0.001) younger than participants who did not respond to the invitation to take part in HIMS.

The sociodemographic, lifestyle and clinical characteristics of men who completed the HIMS assessment are summarised in table 3. Men with schizophrenia spectrum, depression and alcohol-induced disorders were less likely to be married than men with no severe mental disorder, whereas those with depression and alcohol-induced disorders were less likely to have completed high school education. Men with history of alcohol-induced disorders were less physically active than controls without a severe mental disorder. More people with schizophrenia spectrum and alcohol induced disorders were underweight, whereas more people with bipolar disorder were obese. There were more past and current smokers among men with history of schizophrenia spectrum and alcohol-induced disorder, and more current smoking among those with bipolar disorder. Regular alcohol use was less frequent in men with history of depression, but much more frequent among those with a history of alcohol-induced disorder. Two men with history of alcohol-induced disorder denied having previously consumed alcohol. Chronic respiratory illnesses were more prevalent in men with schizophrenia spectrum disorders, coronary heart disease in men with past bipolar, depression and alcohol-induced disorder, and strokes in men with schizophrenia spectrum, depression and alcohol induced disorder. Cox regression showed that the mortality hazard associated with a past severe mental disorder was 2.3 (95%CI = 1.8, 2.9) for schizophrenia spectrum, 1.8 (95%CI = 1.2, 2.8) for bipolar, 1.9 (95%CI = 1.7,2.3) for depression and 2.7 (95%CI = 2.2,3.2) for alcohol-induced disorder. After adjusting the analyses for all the variables listed in the table 3, the respective mortality hazards were 1.9 (95%CI = 1.5, 2.4), 1.5 (95%CI = 0.9, 2.4), 1.6 (95%CI = 1.4, 1.9) and 1.9 (95%CI = 1.6, 2.3). If alcohol use is excluded from the model, the mortality hazards were 2.0 (95%CI = 1.5, 2.5) for schizophrenia spectrum, 1.4 (95%CI = 0.9, 2.3) for bipolar, 1.7 (95%CI = 1.4, 1.9) for depression, and 2.0 (95%CI = 1.7, 2.5) for alcohol-induced disorder.

**Table 3:** Characteristics of older men according to recorded diagnoses of severe mental disorders before the clinical assessment for HIMS.

		No disorder	Schizophrenia-S		Bipolar		Depression		Alcohol	
		N = 11619	N = 97		N = 28		N = 236		N = 154	
Characteristics at assessment:		n (%)	n (%)	OR (95%CI)	n (%)	OR (95%CI)	n (%)	OR (95%CI)	n (%)	OR (95%CI)
Age in years:	65–69	4448 (38.3)	33 (34.0)	Reference	14 (50.0)	Reference	77 (32.6)	Reference	65 (42.2)	Reference
	70–74	4058 (34.9)	39 (40.2)	1.3 (0.8,2.1)	5 (17.9)	0.4 (0.1,1.1)	86 (36.4)	1.2 (0.9,1.7)	58 (37.7)	1.0 (0.7,1.4)
	75–79	2435 (21.0)	17 (17.5)	0.9 (0.5,1.7)	8 (28.6)	1.0 (0.4,2.5)	51 (21.6)	1.2 (0.8,1.7)	25 (16.2)	0.7 (0.4,1.1)
	80+	678 (5.8)	8 (8.3)	1.6 (0.7,3.5)	1 (3.6)	0.5 (0.1,3.6)	22 (9.3)	**1.9 (1.2,3.0)**	6 (3.9)	0.6 (0.3,1.4)
Australian born		5229 (45.0)	38 (39.2)	0.8 (0.6,1.2)	13 (46.4)	1.1 (0.5,2.2)	109 (46.2)	1.0 (0.8,1.4)	80 (51.9)	1.3 (1.0,1.8)
Marital status: married		9533 (82.1)	61 (62.9)	**0.4 (0.2,0.6)**	20 (71.4)	0.5 (0.2,1.2)	166 (70.3)	**0.5 (0.4,0.7)**	85 (55.2)	**0.3 (0.2,0.4)**
Education: high school or above		4698 (40.5)	37 (38.1)	0.9 (0.6,1.4)	14 (50.0)	1.5 (0.7,3.1)	74 (31.4)	**0.7 (0.5,0.9)**	50 (32.5)	**0.7 (0.5,1.0)**
Body mass index:	normal	3540 (30.5)	25 (25.8)	Reference	7 (25.0)	Reference	78 (33.0)	Reference	49 (32.0)	Reference
	underweight	74 (0.6)	3 (3.1)	**5.7 (1.7,19.4)**	0	–	3 (1.3)	1.8 (0.6,6.0)	5 (3.3)	**4.9 (1.9,12.6)**
	overweight	5913 (50.9)	56 (57.7)	1.3 (0.8,2.2)	8 (28.6)	0.7 (0.2,1.9)	109 (46.2)	0.8 (0.6,1.1)	72 (47.1)	0.9 (0.6,1.3)
	obese	2085 (18.0)	13 (13.4)	0.9 (0.4,1.7)	13 (46.4)	**3.1 (1.3,7.9)**	46 (19.5)	1.0 (0.7,1.4)	27 (17.6)	0.9 (0.6,1.5)
Physically active		1979 (17.0)	13 (13.4)	0.8 (0.4,1.4)	5 (17.9)	1.1 (0.4,2.8)	29 (12.3)	0.7 (0.5,1.0)	15 (9.7)	**0.5 (0.3, 0.9)**
Smoking history:	never	3438 (29.6)	16 (16.5)	Reference	9 (32.1)	Reference	68 (28.8)	Reference	18 (11.7)	Reference
	past	6965 (59.9)	65 (67.0)	**2.0 (1.2,3.5)**	9 (32.1)	0.5 (0.2,1.2)	135 (57.2)	1.0 (0.7,1.3)	83 (53.9)	**2.3 (1.4,3.8)**
	current	1216 (10.5)	16 (16.5)	**2.8 (1.4,5.7)**	10 (35.7)	**3.1 (1.3,7.7)**	33 (14.0)	1.4 (0.9,2.1)	53 (34.4)	**8.3 (4.9,14.3)**
Alcohol use:	never	694 (6.2)	6 (6.6)	Reference	0	–	23 (10.0)	Reference	2 (1.3)	Reference
	past	1238 (11.1)	15 (16.5)	1.4 (0.5,3.6)	3 (11.5)	Reference	54 (23.6)	1.3 (0.8,2.2)	41 (27.5)	**11.5 (2.8,47.7)**
	≤ 28 drinks/week	8732 (78.1)	64 (70.3)	0.8 (0.4,2.0)	23 (88.5)	1.1 (0.3,3.6)	145 (63.3)	**0.5 (0.3,0.8)**	75 (50.3)	3.0 (0.7,12.2)
	>28 drinks/week	522 (4.7)	6 (6.6)	1.3 (0.4,4.1)	0	–	7 (3.1)	**0.4 (0.2,0.9)**	31 (20.8)	**20.6 (4.9,86.5)**
Chronic respiratory illness		2520 (22.5)	34 (37.4)	**2.1 (1.3,3.1)**	10 (38.5)	2.1 (1.0,4.7)	56 (24.4)	1.1 (0.8,1.5)	36 (24.2)	1.1 (0.7,1.6)
Hypertension		9523 (82.0)	71 (73.2)	**0.6 (0.4,0.9)**	19 (67.9)	0.5 (0.2,1.0)	182 (77.1)	0.7 (0.5,1.0)	124 (80.5)	0.9 (0.6,1.4)
Diabetes		1322 (11.4)	12 (12.4)	1.1 (0.6,2.0)	5 (17.9)	1.7 (0.6,4.5)	38 (16.0)	**1.5 (1.1,2.1)**	27 (17.5)	**1.7 (1.1,2.5)**
Coronary heart disease		2016 (18.0)	20 (22.0)	1.3 (0.8,2.1)	9 (34.6)	**2.4 (1.1,5.4)**	80 (34.9)	**2.4 (1.9,3.2)**	39 (26.2)	**1.6 (1.1,2.3)**
Stroke		805 (7.2)	13 (14.3)	**2.1 (1.2,3.9)**	4 (15.4)	2.3 (0.8,6.8)	43 (18.8)	**3.0 (2.1,4.2)**	19 (12.7)	**1.9 (1.2,3.1)**
Died during follow up		5245 (45.1)	73 (75.3)	**3.7 (2.3,5.9)**	19 (67.9)	**2.6 (1.2,5.7)**	156 (66.1)	**2.4 (1.8,3.1)**	114 (74.0)	**3.5 (2.4,5.0)**

Schizophrenia-S: schizophrenia spectrum disorders. See text for information about the mortality hazard. OR: odds ratio, 95%CI: 95% confidence interval of the odds ratio.

## Discussion

The recorded prevalence of severe mental disorders in this large community-representative sample of older men was 5.8%. Schizophrenia spectrum disorders affected 1.2% of the population, bipolar 0.3%, depression 2.5%, and alcohol-induced disorders 1.8%. The mortality hazard ratio for men with a severe mental disorder was 2.3 times greater than for men free of significant mental health problems, and this excess mortality occurred across all four diagnostic groups under investigation. The mortality rate increased with increasing age, and the gap in the rates between men with and without history of severe mental disorder remained more or less stable for the different age groups. Our data also showed that men with severe mental disorders who agreed to complete a clinical assessment had different educational, sociodemographic, lifestyle and clinical background, although these variables could not adequately explain the excess mortality of this population.

### Strengths and limitations of the study design

This study has the merit of having assembled a large community-representative sample of older men, which allowed us to ascertain with precision the unbiased prevalence of relatively uncommon mental disorders of older age. Establishment of ‘caseness of mental disorder’ was based on data retrieved from the WADLS since 1966, which covers all inpatient and outpatient contacts with mental health services (but not primary care) [26]. We have treated our prevalence estimates as indicative of the lifetime presence of the disorder, although health contacts occurring exclusively before 1966 would have been missed. This could have generated ‘false-negative’ cases, although the chronic and recurring course of these disorders suggest this is unlikely to have been a source of bias. Even it that were the case, the potential inclusion of cases among men assigned to the ‘no severe mental disorder’ group would have minimised differences in mortality hazard when we compared people with and without severe mental disorders. Hence, the observed differences are most likely valid, although we cannot dismiss the possibility that the effect of severe mental disorders on mortality could be higher than our estimates suggest. We also acknowledge that case-ascertainment using record linkage may not be as accurate or as valid as structured systematic assessments that lead to a diagnosis according to accepted criteria. Existing data suggest that the WADLS diagnoses for severe mental disorders such as schizophrenia and bipolar disorder are valid [20]. The diagnosis of alcohol-induced disorder has some face-validity regarding mortality [7], and our finding that 1 in every 5 men who had received this diagnosis were still drinking heavily at the time of assessment for HIMS suggest that they had been, and perhaps some had continued to be, problem drinkers.

Every person deceased in Australia is entered in the death registry, which forms part of WADLS. The registry allows for the recording of the main cause of death, as well as up to 20 other secondary causes. In this study, we used only the code for the main cause of death and concede that there is a certain degree of subjectivity on how these diagnoses are assigned. For example, a person with schizophrenia might be admitted to hospital with a chest infection that eventually leads to death. Some doctors may record schizophrenia as the main cause of death (particularly if the chest infection is considered to have arisen as a result of poor mobility associated with the illness), while others might record infection. For this reason, we included substance-induced and other mental disorders as possible causes of death in this cohort. It is also conceivable that some of the men included in the study moved away from Australia and that their deaths were not recorded in the death registry. However, movements away from Australia are minimal in this older age group [27], suggesting that this would have had a negligible impact on our estimates. Importantly, our analysis of the causes of death took into account competing risks, and this approach to the analysis of the data provides a more accurate estimate of the risk of death due to a specified condition than Cox regression.

The inclusion of prevalent cases of dementia in the sample could have biased our results, as these patients may present psychiatric and behavioural symptoms that could be misdiagnosed as a psychotic or mood disorder. Such misclassification bias would have contributed to increase the mortality hazard of people with a severe mental disorder, as older people with dementia have lower life expectancy than those without [28]. We tried to minimise such a possibility by excluding from the study 588 people with a recorded diagnosis of dementia (known prevalent cases) and by completing a sensitivity analysis that excluded men who had died within the initial two years of follow up: the results remained virtually unchanged. This sensitivity analysis also aimed to decrease confounding due to severe prevalent medical morbidity when follow up started. For example, if the diagnosis of a severe mental disorder (e.g., depression) were made opportunistically at the time of admission to hospital because of a significant medical morbidity (e.g., stroke), then short-term survival ascribed to the mental disorder would have been confounded by the concurrent morbidity.

Moreover, we acknowledge that we did not have access to data about factors likely to mediate or facilitate the premature death of all older men with severe mental disorders. This limitation was partly circumvented by analysing the data from the 12136 HIMS participants, which showed that some sociodemographic, lifestyle and clinical factors could potentially attenuate the risk of death among these men. We concede, however, that HIMS was associated with healthy participant bias and that inferences drawn from this sample should be interpreted with caution. Finally, it is important to note that the results of this study were limited to men and may not be directly applicable to women, although the results of other investigations indicate that the life expectancy of women with severe mental disorders is similarly reduced [2].

### Interpretation of the findings

The results of this study confirmed our prediction that the recorded lifetime prevalence of severe mental disorders in later life would be lower than that reported for younger age-groups. We found that the prevalence of severe mental disorders in this old cohort of men was 5.8%. The Health 2000 Study recruited 8028 people aged 30 years or older living in Finland [29]. After an initial screening for psychotic symptoms, eligible participants were interviewed with the Composite International Diagnostic Interview (CIDI) and lifetime psychiatric diagnoses followed the DSM-IV criteria. Non-affective psychosis (akin to our definition of schizophrenia spectrum disorder) was present in 2.3% of those aged 45–54, 55–64 and over 65 years [29]. The prevalence of bipolar disorder decreased from 0.4% among those aged 45–54 to 0.2% among those aged 55 to 64 and 0.1% among those older than 65 years, although confidence limits were wide and consistent with our results [29]. In the present study, it is conceivable that false positive cases were included in the group of schizophrenia spectrum disorders because of the hierarchical approach we used to ascribe diagnosis. The consequence of such a misclassification would be inflation in the prevalence of schizophrenia spectrum disorders relative to other diagnostic categories, such as bipolar disorder.

The prevalence of depressive disorders in our study was lower than the lifetime prevalence of 10.6% among those aged over 60 years reviewed by the National Comorbidity Survey Replication, which in turn was 1.5 to 2 times lower than in younger age-groups [30]. Such a discrepancy may be partly due to the fact that, among the severe mental disorders, major depression is the condition most commonly treated outside mental health specialist settings [31]. Consequently, depressive disorders recorded in the WADLS are most likely true cases (i.e., high specificity), although the system may lack sensitivity to identify uncomplicated depression managed successfully in the primary care sector. In addition, the hierarchical approach we used to ascribe diagnoses (schizophrenia spectrum then bipolar then depressive then alcohol-induced disorders) might have contributed to reduce the number of people identified as having a depressive disorder, and may further explain the relative low prevalence of depression in our sample.

Data from the Australian Survey of Mental Health and Well-Being showed that the 12-month prevalence of alcohol use disorders falls from 10.5% among those aged 18–34 years to 1.8% for people older than 55 years [32]. However, American data suggest that lifetime prevalence in later life may be as high as 16.1% [33]. As for depressive disorders, the hierarchical system that we used to assign diagnoses could have decreased the number of men with alcohol-induced disorders, as some of them might have been ascribed other diagnoses (schizophrenia spectrum, bipolar or depression) [34]. This, together with the limited sensitivity of the WADLS to record uncomplicated cases (for example, alcohol abuse), may have led to an underestimation of the true lifetime prevalence of alcohol-induced disorders in the present cohort. Notwithstanding these potential caveats, the WADLS identified cases of severe mental disorder that require assistance from specialist medical services. We acknowledge, though, that we did not have access to comparable data from other age groups and that our inferences about the decreasing prevalence of severe mental disorders in later life are indirect.

Our data also confirmed that people with recorded history of past severe mental disorders who reach old age have lower life expectancy and greater mortality hazard than men with no such history. As a group, men with a past severe mental disorder died 2.8 years earlier than their counterparts and were twice as likely to die during the subsequent 14 years of follow up. The underlying reasons for this excess mortality are not immediately apparent, although others have suggested that hazardous lifestyle (such as smoking) and poor physical health drive this process [10]. Using data from a subsample of this cohort (HIMS), we found that men with schizophrenia spectrum, bipolar, depression and alcohol-induced disorders differed from their counterparts on a number of lifestyle, medical, educational and sociodemographic variables, but that the excess mortality of those with mental disorders could not be explained entirely by these factors (although statistical adjustment for these factors did attenuate the hazard ratio). It is also possible that the severity of the medical comorbidities among those with mental disorders was different. For example, diabetes may be present in both groups, but may not be as well controlled in men with a severe mental disorder. There is evidence that people with severe mental disorders do not receive the same level of care for physical comorbidities as people without these disorders [35], and their compliance with medical treatments may be suboptimal [36]. Thus, people with severe mental disorders may have more frequent medical comorbidities which, when present, may be more severe than in the general population because of suboptimal access to services and compliance with treatments. This could explain why medical complications and death may occur earlier in older people with than without severe mental disorders. As a result, we have had to reject our hypothesis that men with severe mental disorders who reach older age do not have greater mortality than their peers. In fact, out data indicate that the unfavourable clinical outcomes seen in younger patients with severe mental disorders persist later in life. Whether appropriate treatment with psychotropic medications would decrease or increase the risk of death of older men with severe mental disorders cannot be determined from our data.

Cardiovascular diseases were the most frequent cause of death of older men with and without severe mental disorders, although there was a significant excess of cardiovascular events among cases. Infections, cardiovascular and respiratory diseases, mental disorders, and diseases of the central nervous system were more frequent causes of death among those with schizophrenia spectrum disorder than men without a severe mental disorder. Cancer was less frequent among the former than the latter (albeit not significantly), and there were no deaths by suicide in the schizophrenia spectrum group. Such a mortality pattern is similar to that observed among younger people with schizophrenia and related disorders: what kills people with schizophrenia spectrum disorders in early life continues to kill those who reach old age [10], [21], [37], [38]. The exception, in the case of schizophrenia spectrum disorders, is suicide, which is not infrequent in early life [39]. The number of men with bipolar disorder in the sample was small, which contributed to the imprecision of the estimates for cause of mortality. Nonetheless, the excessive mortality due to respiratory diseases is noteworthy, particularly in light of the high prevalence of smoking [40]. Two men with bipolar disorder died by suicide, which is also a more frequent cause of death in younger people with bipolar disorder than in the general population [41]. Depression was associated with excessive mortality due to infections, cancer, cardiovascular and respiratory diseases, as well as other causes. These findings are consistent with those reported for younger people with depression [42]. Similarly, men with a recorded history of alcohol-induced disorder had higher risk of dying as a result of infections, cancer, cardiovascular and respiratory diseases [43]. Taken together, these results show the causes of death associated with schizophrenia spectrum, bipolar, depression and alcohol-induced disorders overlap, although differences in the distribution of deaths attributable to cancer and suicide seem to differ.

## Conclusions

This study has shown that older men with severe mental disorders have greater mortality hazard and lower life expectancy than their peers. The underlying reasons for this increase in mortality are not entirely clear, but hazardous lifestyle choices, the presence of disabling medical comorbidities, poor compliance with treatment, and suboptimal access to health services may all play a part. There is now evidence that adoption of healthy lifestyle practices, even very late in life, is associated with measurable health benefits, so that smoking cessation, limited alcohol consumption and physical activity should become an integral part of the management of older adults with severe mental disorders [44], [45]. Similarly, the management of chronic medical conditions, such as hypertension, can decrease the risk of adverse health events among octogenarians, which indicates that there is no reason to withhold effective treatments from older people when these are available.

As a community, we are yet to address successfully the excess mortality associated with severe mental disorders. Unlike young adults, most older people will be in contact with health services at least once a year [46], offering policy makers and health professionals a unique opportunity to intervene and improve the clinical outcomes of this segment of the population. Perhaps these older adults could teach us important lessons that would eventually guide the design of interventions that are effective at improving the clinical outcomes of people with severe mental disorders across the lifespan.
